# Pollination in a new climate: Assessing the potential influence of flower temperature variation on insect pollinator behaviour

**DOI:** 10.1371/journal.pone.0200549

**Published:** 2018-08-01

**Authors:** Mani Shrestha, Jair E. Garcia, Zoë Bukovac, Alan Dorin, Adrian G. Dyer

**Affiliations:** 1 School of Media and Communication, RMIT University, Melbourne, Australia; 2 Faculty of Information Technology, Monash University, Melbourne, Australia; 3 Department of Physiology, Monash University, Melbourne, Australia; Indian Institute of Science, INDIA

## Abstract

Climate change has the potential to enhance or disrupt biological systems, but currently, little is known about how organism plasticity may facilitate adaptation to localised climate variation. The bee-flower relationship is an exemplar signal-receiver system that may provide important insights into the complexity of ecological interactions in situations like this. For example, several studies on bee temperature preferences show that bees prefer to collect warm nectar from flowers at low ambient temperatures, but switch their preferences to cooler flowers at ambient temperatures above about 30° C. We used temperature sensor thermal probes to measure the temperature of outdoor flowers of 30 plant species in the Southern regions of the Australian mainland, to understand how different species could modulate petal temperature in response to changes in ambient temperature and, potentially, influence the decision-making of bees in the flowering plant’s favour. We found that flower petal temperatures respond in different ways to changing ambient temperature: linearly increasing or decreasing relative to the ambient temperature, dynamically changing in a non-linear manner, or varying their temperature along with the ambient conditions. For example, our investigation of the difference between ambient temperature and petal temperature (*ΔT*), and ambient temperature, revealed a non-linear relationship for *Erysimum linifolium* and *Polygala grandiflora* that seems suited to bee temperature preferences. The temperature profiles of species like *Hibertia vestita* and *H*. *obtusifolia* appear to indicate that they do not have a cooling mechanism. These species may therefore be less attractive to bee pollinators in changing climatic conditions with ambient temperatures increasingly above 30° C. This may be to the species’ detriment when insect-pollinator mediated selection is considered. However, we found no evidence that flower visual characteristics used by bees to identify flowers at close range, such as colour or shape, were straightforward modulators of floral temperature. We could not identify any clear link to phylogenetic history and temperature modulation either. Mapping our test flower distribution on the Australian continent however, indicates a potential clustering that suggests different flower responses may constitute adaptations to local conditions. Our study proposes a framework for modelling the potential effects of climate change and floral temperature on flower pollination dynamics at local and global scales.

## Introduction

Changes in flowering phenology and potential changes in climatic conditions may hold important implications for plant traits including leaf emergence, flowering time, and seed germination [1,2]. Climate change could also have consequences influencing geographical distributions, and local abundances. It may also disturbing the phenology of flowering plants, potentially creating spatio-temporal disruptions of their interactions with essential pollinators [2–4]. Currently, interest has mainly focused on potential mismatches between flower and pollinator emergence times [5–11]. A few studies [12–14] have considered the plasticity of plants or pollinators to potentially adapt to changing conditions, the extent to which this may alter fitness. Global temperature variation has shifted flowering time and the magnitude of variable phenological responses between different species [14–16]. For example, Galloway and Burgess [4,17] showed that changes in flowering date may affect subsequent reproductive traits of offspring by reducing seed set, seed size, composition, and dormancy, as well as the time of seed dispersal. Richardson et al [14] found some evidence of plasticity for flowering phenology in the big sagebrush (*Artimesia tri-dentata*) from Idaho and Utah USA. They suggested that this plant may accommodate changes in flowering date up to about two weeks, a change predicted to be plausible within the 21^st^ century. However, there remains a paucity of data on how changes like these, and others plausibly resulting from climate change, may influence plant-pollinator interactions around the world.

Insects are among the most important pollinators of flowering plants–many plants rely solely on insects for successful reproduction [18–22]. Flowering plants often attract pollinators by providing nectar [23,24], pollen [24,25], and/or thermal rewards [26–30], using multimodal signals including colour [31–38] and shape [39,40] or a scent component [34,41–43]. The recent publication of the Food and Agriculture Organisation (FAO) of the United Nations on ‘Potential effects of climate change on crop pollination’ reports that insect pollination is vital for about 35% of global food production for human consumption [44–46]. These studies estimate the value of insect pollination to the world economy to be in the range of 235–577 billion US$/year [47]. The Intergovernmental Panel on Climate Change [48] has published data suggesting a likely increase in global temperature in the range of 1.1 to 6.4°C by the end of this century, and more recent reports have confirmed predicted temperature rises within this range [49]. However, the complexity of global climate change means that whilst some regions may warm, other areas may cool in a dynamic way, often influenced by major ocean currents that drive weather patterns [48,50]. Thus, there may be a need for plants to adapt to changing local climatic conditions that influence important plant-pollinator relationships in complex and unanticipated ways.

Honey bees, bumble bees and stingless bees are major pollinators of many important agricultural crops and plants in natural ecosystems. Studies have shown that bees often prefer thermal rewards like warm flowers and warm nectar [28,51,52]. However, their preferences change towards cooler nectar when ambient temperature increases to above ca. 30° C [52]. Specifically, [52] showed using thermal imaging that observed bee thermal preferences are due to a need to regulate body temperature during foraging. This is likely to be due to the requirement of bees to have a thoracic temperature around 30° C for flight [26]. Interestingly, studies of alpine plants suggest that flowers can accumulate heat on petal surfaces, which are then significantly warmer than canopy foliage on bright days [53]. A study by [54] found that sub-Antarctic megaherbs *Pleurophyllyum speciosum* exhibited higher leaf and floral temperatures than ambient temperature (leaves +9° C, inflorescence +11° C compared to ambient temperature). Both studies [53,54] thus suggested that insect pollinators could benefit from thermal rewards produced by flowers, which would help them to reduce the energy cost associated with flying in cool ambient temperatures [26,27,51,52,55,56].

Studies have shown that different plant species can modulate their temperature using various mechanisms. For example, heliotropism (sun tracking: [30,57–59]), thermogenesis [60] and changing morphological features such as colour and shape [53,54,56,61–63] can increase intra-floral temperature to levels up to 11°C above ambient conditions. In very warm conditions, some plants may also modulate flower temperature through evaporative cooling or self-shading [64], while other plants have been shown to tightly regulate the temperature within a 2°C range using a combination of warming and cooling mechanisms [65]. These phenomena may hold implications for plant-visiting ectothermic insects [60,66–69]. Given that flower temperature can directly influence pollinator temperature when nectar is imbibed [52], the use of temperature coupled with a nutritional reward may influence the relative success of flower pollination with fitness benefits for certain plant types depending upon localized ambient temperature conditions [28,52]. Currently, however, there is a lack of information on how floral traits may interact with temperature, and whether flower temperature modulation might only be specific to extreme environments.

In the current study, we surveyed a wide variety of flowering plants with different morphologies, colours and plant forms including native and naturalized alien species to Australia. Australia is a valuable test example as flowers have been shown to have independently evolved signals like colour to valuable pollinators such as bees [32,35], and the distribution of floral spectra is consistent with worldwide flowering signal evolution [32,33,35,70]. In particular, we were interested in understanding potential temperature modulation in a range of ambient environments that occur in the temperate region of Australia. We interpret the analyses of our temperature data for flowering plants in relation to a model of native Australian bee responses to feeding from sucrose at different temperatures, depending upon the ambient temperature. Our survey provides an understanding of the variation of flower temperature with respect to ambient temperature and floral traits. Thus, we seek to test if all flowers in this temperate environment conform to similar patterns of warming/cooling relative to ambient temperature, across a diversity of weather, sunlight, moisture and soil conditions likely to be encountered by insect pollinators, or if there are distinct floral relationships to temperature that might potentially benefit certain plant species in specific regions as climatic conditions change.

## Materials and methods

### Data collection

#### Sample species

**Fig 1:**
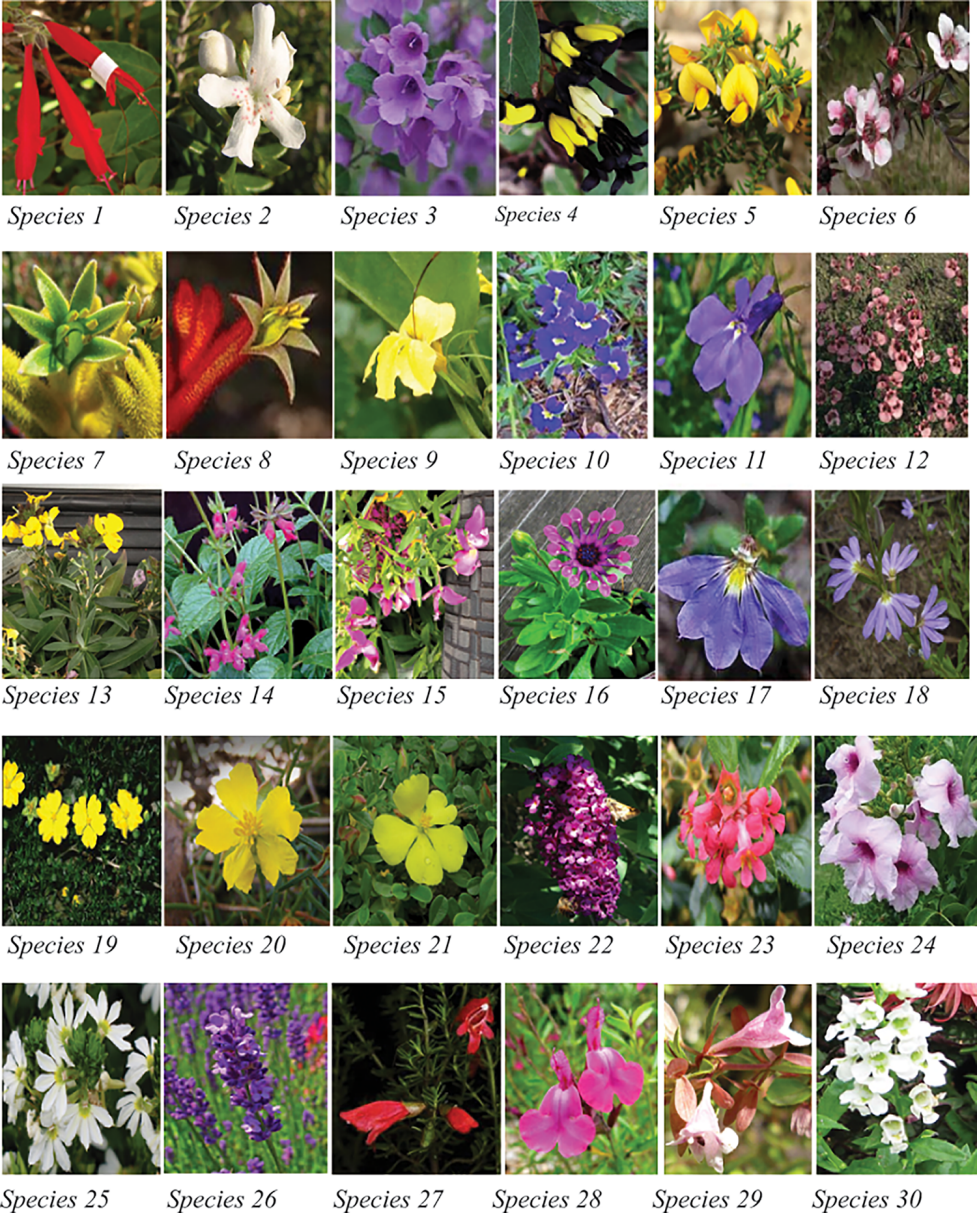
Colour images of the flowering plants used in the current experiment. Species names appear in Table 1.

We conducted our experiment at Monash University, Clayton Campus from late August 2013 to late January 2014 covering Australian winter, spring and summer. Melbourne is located in the temperate region of Australia with daytime temperatures ranging from about 7° C in winter to more than 40° C in summer. We selected 30 different plant species that are found in this temperate environment (Table 1, Fig 1) including some species that were grown at Monash University as well as species supplemented from local nurseries. Flowers were selected to be generally representative of a range of sizes, shapes and colours, traits that might affect their capacity to modulate temperature. In addition, the colour spectra of these flowers broadly represent known flower distributions for Australia and globally (see results). Flower longevity, the length of time a flower remains open and functional, differed between flower types and varies among plant species [71] In our sample, flower longevity varied from 4 or 5 hours (e.g. *Pultenaea graveolens*) to many days in some cases (e.g. *Scaevola* spp., *Prostanthera* spp.) (Table 1). Most of the selected species present flowers with a single colour distributed roughly uniformly over the entire flower (e.g. Fig 1). However, some species such as Sp. 4, show two dominant colours. In this case, the two colours are treated separately in our experiment.

**Table 1:** Effect of ambient temperature on *ΔT* for the 30 sampled flower petals. Fit type indicates the kind of model that best fitted the data: non-linear (GAM) or linear (GLS). Significance of all models was evaluated at α = 0.05. Details of each fit are available in Figs 5–10. Flower shape is abbreviated as Boat shape (BS), Open (O), Open tubular (OT), tubular (T) and flower colours in the hexagon colour space are abbreviated as *BLUE* (G), *BLUE-GREEN* (BG), *GREEN* (G), *UV-GREEN* (UG), *ULTRAVIOLET* (UV) and *UV-BLUE* (UB), as defined by [74]. Colour in the table represents non-significant (NS, green), significant non-linear relationship (magenta), significant linear positive (warm-yellow) and significant linear negative (cool-blue). * indicates a short flowering time (ca. 5–9 hours). Species 4 has two parts (4a and 4b) measured and analysed individually.

			95% CI for coefficients for linear cases		Flower shape	
Species name	Fit type	P-value	Intercept	Slope		Hue
*Salvia sp*. (1)	Non-linear	0.0005	NA	NA	T	B
*Westringia fruticosa* (2)	Non-linear	0.0005	NA	NA	O	BG
*Prostanthera melissifolia* (3)	Linear	**< 0.05**	-7.23, -1.95	0.240, 0.599	T	B
*Kennedia nigricans* (black) (4a)	Linear	**< 0.05**	5.36, 14.8	-0.531, -0.118	BS	G
*Kennedia nigricans* (yellow) (4b)	Linear	**< 0.05**	-0.904, -3.47	0.26, 0.570	BS	UB
^*^Pultenaea graveolens (5)	Linear	**< 0.05**	-27.2, -9.8	0.50, 1.31	BS	UG
*Leptospermum sp*. (6)	Linear	**< 0.05**	4.52, 11.21	-0.621, -0.206	O	B
*Anigozanthos manglesii* (7)	NS	> 0.05	-10.13, 3.45	-0.122, 0.860	T	B
*Anigozanthos flavidus* (8)	NS	> 0.05	0.309, 3.25	-0.044, 0.082	T	BG
*Goodenia ovata* (9)	Non-linear	0.0005	NA	NA	O	UG
*Dampiera diversifolia* (10)	Non-linear	0.003	NA	NA	O	B
*Lobelia ensifolia* (11)	Non-linear	0.0005	NA	NA	O	B
*Diascia barberae* (12)	Linear	**< 0.05**	-2.08, -0.745	0.095, 0.145	OT	UB
^*^Erysimum linifolium (13)	Non-linear	0.0005	NA	NA	OT	G
*Salvia chiapensis* (14)	Non-linear	0.01	NA	NA	T	B
*Polygala grandiflora* (15)	Non-linear	0.0005	NA	NA	BS	UB
*Osteospermum ecklonis* (16)	Linear	**< 0.05**	1.94, 4.72	-0.207, -0.069	O	B
*Scaevola aemula* (purple) (17)	Linear	**< 0.05**	-2.03, -0.337	0.092,0.165	O	B
*Scaevola albida* (18)	Linear	**< 0.05**	-0.532, 0.803	0.012, 0.061	O	BG
^*^Hibbertia pedunculata (19)	Linear	**< 0.05**	-3.14, -2.48	0.084, 0.138	O	UG
^*^Hibbertia vestita (20)	Linear	**< 0.05**	-3.40, -2.125	0.164, 0.231	O	UG
^*^Hibbertia obtusifolia (21)	Linear	**< 0.05**	-3.82, -3.29	0.208, 0.244	O	UG
*Buddleja davidii* (22)	Non-linear	0.0005	NA	NA	T	B
*Escallonia macrantha* (23)	NS	> 0.05	0.504, 1.42	-0.029, 0.041	OT	BG
*Pandorea jasminoides* (24)	Linear	**< 0.05**	-1.82, -0.318	0.077, 0.136	OT	BG
*Scaevola aemula* (white) (25)	Non-linear	0.0005	NA	NA	O	BG
*Lavandula angustifolia* (26)	Non-linear	0.001	NA	NA	T	B
*Prostanthera aspalathoides* (27)	Linear	**< 0.05**	-5.69, -1.43	0.086, 0.289	T	BG
*Salvia greggii* (28)	NS	> 0.05	-0.528, 2.91	-0.039, 0.056	T	BG
*Abelia schumannii* (29)	Linear	**< 0.05**	-7.47, -5.33	0.300, 0.386	T	NA
*Angelonia angustifolia* (30)	Linear	**< 0.05**	-3.36, -1.61	0.037, 0.122	O	BG

We classified the flowers’ morphology as boat shape (BS), open (O), open tubular (OT), and tubular (T) (Fig 1, Table 1), following the standards presented in the taxonomical literature [72,73].

#### Flower colour measurement

We measured flower colour of the experimental species using an Ocean Optics spectrophotometer (Dunedin, FL, USA, 2011) following the methods employed by [32] and [35]. To understand flower colour perception based on hymenopteran pollinators we categorized colour into six categories: ‘*BLUE*’ (G), ‘*BLUE-GREEN*’ (BG), ‘*GREEN*’ (G), ‘*UV-GREEN*’ (UG), ‘*ULTRAVIOLET*’ (UV) and ‘*UV-BLUE*’ (UB), as defined in the hexagon colour space by [74].

#### Flower temperature measurement

The experiment used a thermocouple typically employed to measure either insect body temperature or flower temperature [28,53,54,61,75]. A Voltage Sensor Data Logger (VSL) was used to record petal temperature measurements. The data logger enabled us to run experiments continuously using PC-managed software. The VSL was supplied by ICT International PTY LTD (NSW, Australia, 2013, equipment no: VSL1D702) and equipped with an Automatic Weather Station (AWS) to measure humidity and ambient temperature, a Light Sensor (PAR) to measure incident light, and a Temperature Sensor Meter (TSM) to record the flower temperature (in our case each flower is a target object) variation during a day. The TSM contains a THERM-MICRO Thermistor sensor and has ±0.2°C tolerance from 0°C to +70°C that can be operated within a temperature range of -40°C and +80°C.

**Fig 2:**
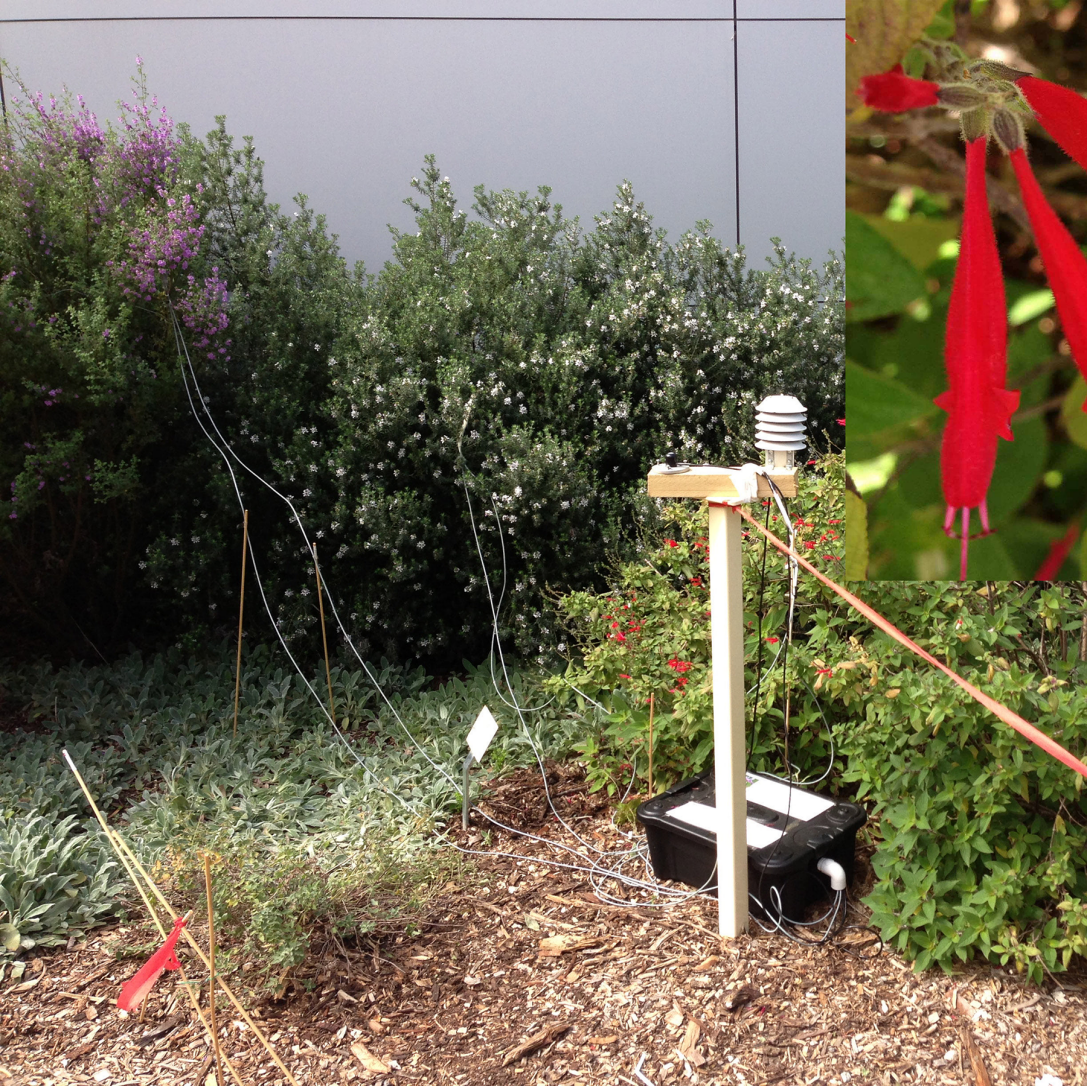
Equipment set up in the field to collect flower temperature readings. Inset shows the TSM sensor attached to a petal surface.

We recorded flower temperature and ambient temperature over periods from five hours to more than 24 hours depending on specific floral longevity (Table 1). TSMs were attached inside and outside petal surfaces (Fig 2). At least 3- and up to -5 flowers were used to measure the flower temperature (Fig 2) for each species. Informed by pilot experiments, we measured the temperature with a fixed sampling rate of 240 seconds using PC-managed software, leaving the equipment in the field during data collection. We did a series of experiments in an open garden. Flower temperature was measured from the broad display part of the flower under the petal surface and away from direct sunlight, in order to reduce the effect of direct sun on the thermocouples (Fig 2). In these experiments we only analyse daytime temperature data from sunrise to sunset.

### Data analysis

#### Temperature data analysis

To understand the potential effect of ambient temperature on petal warming, we defined a response variable (*ΔT*) representing the difference between the ambient and petal temperature for each one of the selected experimental species. We used regression techniques to evaluate the potential effect of ambient temperature on *ΔT* and subsequently to classify the plant species based on this property.

For each one of the measured species, we implemented a power of the covariate (varPower) and exponential (varExp) variance structures to model variance heterogeneity; and, auto-regressive model of order 1 (corAR-1) and moving average (ARMA) correlation structures to account for the time-series resulting from sampling at fixed time intervals [76]. Variance and correlation structures were estimated using restricted maximum likelihood employing the mgcv package [77] available for the *R* environment for statistical computing version 3.3.1 [78]. The best variance and correlation structure combination for each species were selected based on the Alkaike Information Criteria (AIC) for each model and, whenever possible, results were corroborated by likelihood ratio tests [76].

In our regression analyses, we tested for a significant effect of ambient temperature on *ΔT*. We began by fitting a non-linear Generalised Additive Model (GAM) with a Gaussian distribution to the data and subsequently comparing its explanatory power against a simpler, linear model for all the datasets. If the explanatory power of the two models was non-significantly different, we always selected the simpler, linear model over the non-linear alternative following standard methods [76]. If the analysis suggested that a linear relationship was a better model to explain the relationship, we used generalised least squares (GLS) to fit a linear model including the variance and correlation structures for the respective species [76]. The effect of the fixed term was estimated using maximum likelihood following standard protocols. GLS and GAM models were also implemented using the mgcv package.

The presence of a non-linear relationship between ambient temperature and *ΔT* is considered to be evidence for the capacity of a plant species to maintain a target petal temperature which is either cooler or warmer than ambient temperature by more than 1°C. A temperature difference larger than this threshold has been shown to be biologically significant to important insect pollinators such as bumble bees [28,51] and native Australian bees [52].

Bootstrapping with 100,000 replicates was implemented for inferring and constructing 95% confidence intervals for the coefficients of the linear and non-linear models [76,79]. In the former case, confidence intervals were directly estimated from the regression model. For the non-linear models, bootstrap samples were used to obtain precise P-values for the smoother term [79].

After completing the individual regression analysis, we used a Pearson’s chi-square analysis to test for independence between the flower types identified from the regression analysis and the morphological traits of shape and colour.

#### Modelling bee preference for warm nectar reward

**Fig 3:**
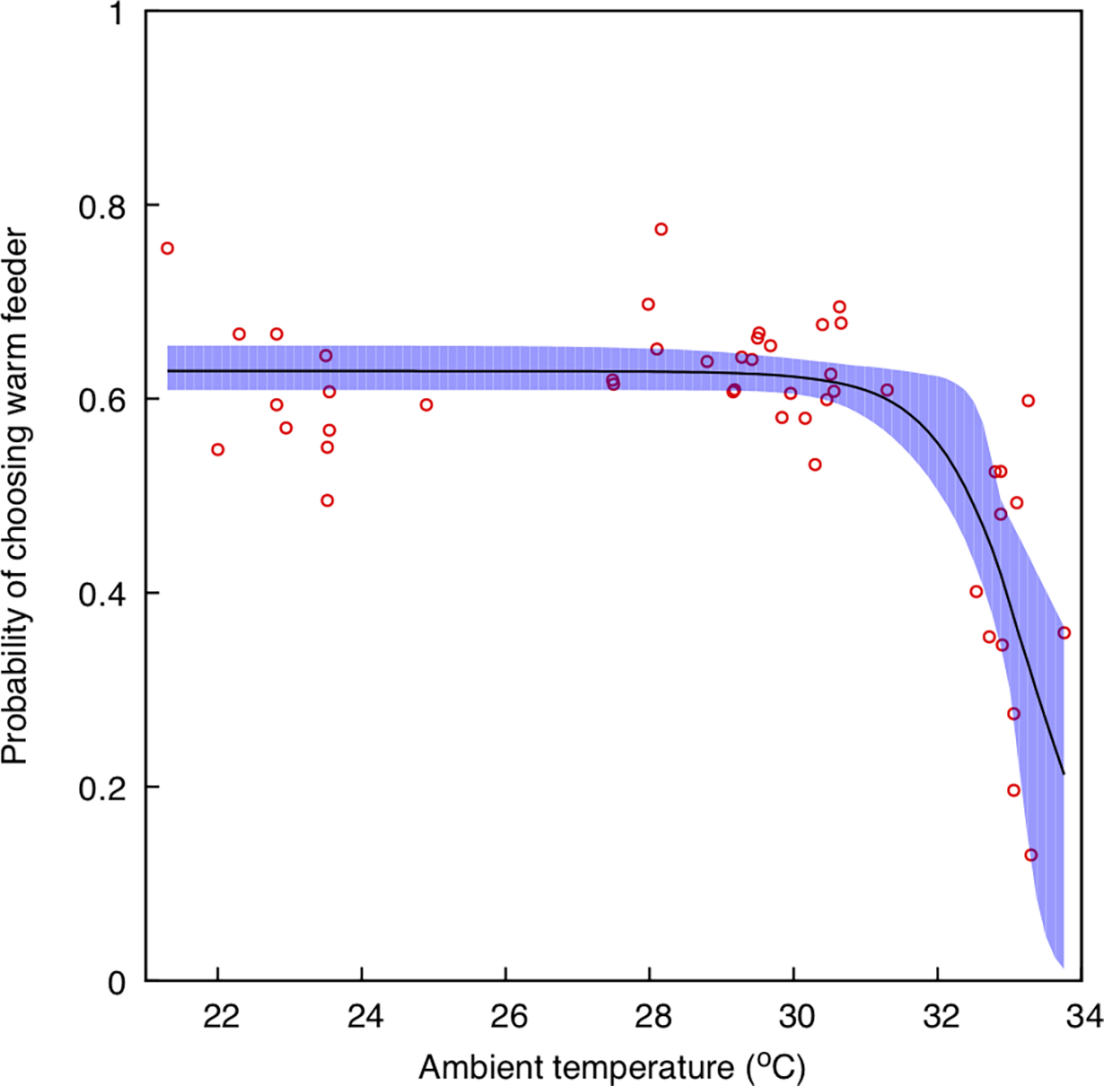
Non-linear, 3-parameter logistic model (solid black) describing the probability of bees choosing a warm nectar feeder rather than an ambient temperature feeder (*y*-axis) at different ambient temperatures (*x*-axis) and 95% confidence region (blue shaded area). Circles represent the data collected by [52].

To interpret the biological significance of modulation of *ΔT* by flowers, it is important to understand bee preferences for flower temperature. To this end, we built a model describing bee preferences as a function of temperature. We used Norgate et al [52] data on Australian native bee (*Tetragonula carbonaria*) preferences for warm nectar rewards at different ambient temperatures (Fig 3) to build the model using a non-linear, 3-parameter logistic function of the form [80]:

$$\pi (T)=\frac{a}{1+{exp}^{\left[(b-T)/c\right]}},$$ (1)

where a, b, and c are constants, and (*π*) is the predicted probability of choosing warm nectar at a given ambient temperature (*T*). The coefficients describing the model of Eq 1 were fitted using generalised least squares with the routine *gnls* available in the package *nlme* version 3.1[77] for the *R* language and environment for statistical computing v 3.4.1 [78]. We bootstrapped the non-linear regression model 100,000 times by pairs to recover the 95% confidence intervals of the coefficients [79,81].

To evaluate the adequacy of the non-linear model with three parameters to capture the observed behavioural data, we compared this model against: i) a linear, generalised linear model (GLMM) with a logit link and assuming a binomial family; and, ii) a simpler, non-linear model with just two parameters. Model comparison was performed using the Alkaike Information Criteria (AIC), whilst the comparison between the two- and three-parameter non-linear models was conducted employing a test of deviance [82].

#### Bee preference warm nectar reward modelling

We used published data [52] to interpret the modulation of ΔT by flowers to understand the bee preferences for flower temperature. We thus constructed a model of bee temperature preferences using data for the Australian native bee *T*. *carbonaria* [52] (details provided in result sections).

## Results

### Flower temperature modelling / Temperature data analysis

**Fig 4:**
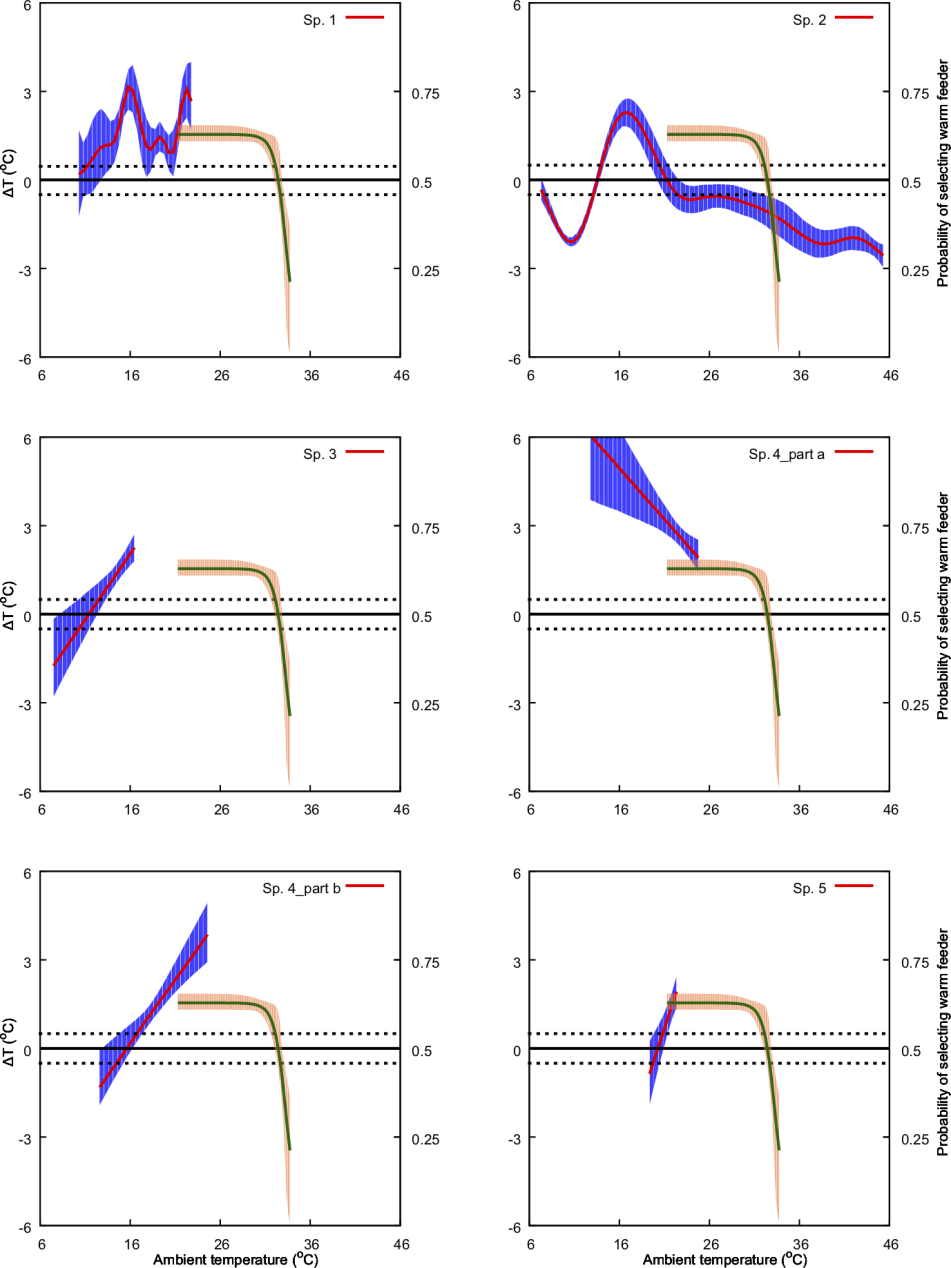
Predicted effect of ambient temperature (*x-*axis) on ΔT (primary *y-*axis: left-hand side) and on bee preference for warmer nectar reward (secondary *y*-axis: right-hand side). The model for ΔT is represented by the solid red line along with its 95% confidence region (shaded blue region). The solid green line represents the preference model along with its 95% confidence region (shaded orange region). Species numbers correspond to those in Table 1. Black, dotted lines indicate ΔT values of -1 and 1°C. Refer to Methods sections for details of the bee preference function.

**Fig 5:**
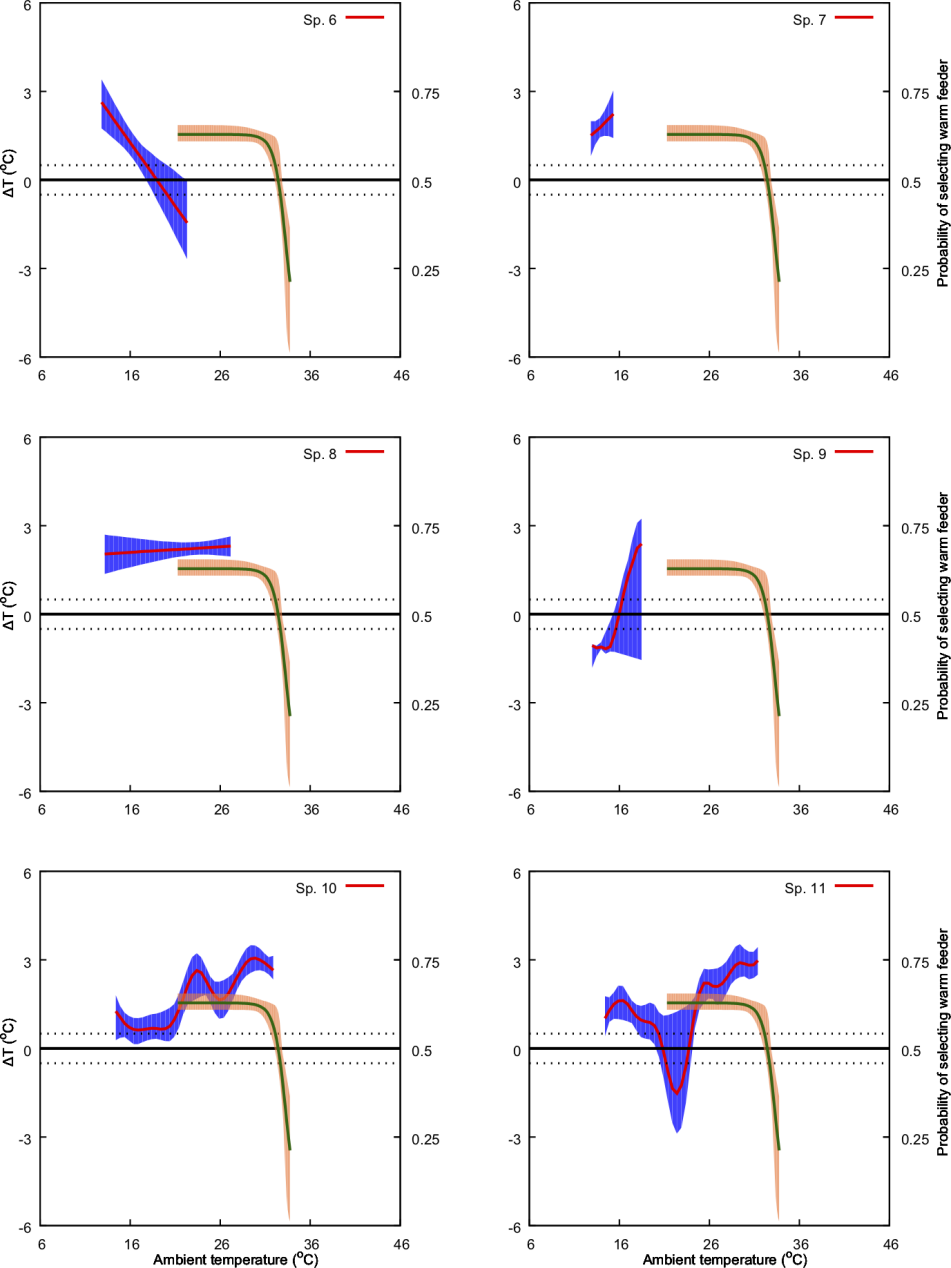
Predicted effect of ambient temperature (*x-*axis) on ΔT (primary *y-*axis: left-hand side) and on bee preference for warmer nectar reward (secondary, *y*-axis: right-hand side). The model for ΔT is represented by the solid red line along with its 95% confidence region (shaded blue region). Solid green line represents the preference model along with its 95% confidence region (shaded orange region). Species numbers correspond to those in Table 1. Black, dotted lines indicate ΔT values of -1 and 1°C. Refer to Methods sections for details of the bee preference function.

**Fig 6:**
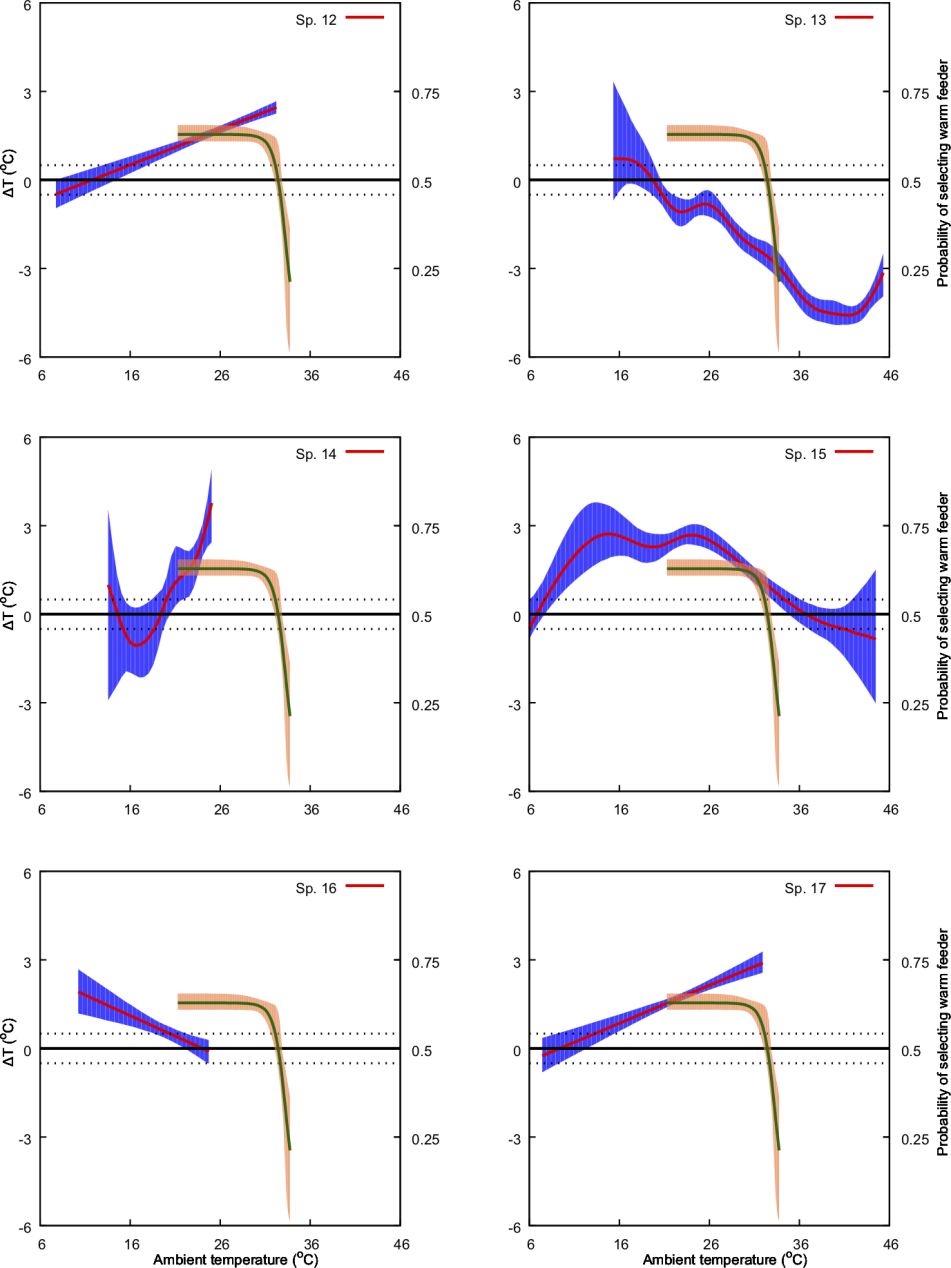
Predicted effect of ambient temperature (*x-*axis) on ΔT (primary *y-*axis: left-hand side) and on bee preference for warmer nectar reward (secondary, *y*-axis: right-hand side). The model for ΔT is represented by the solid red line along with its 95% confidence region (shaded blue region). Solid green line represents the preference model along with its 95% confidence region (shaded orange region). Species numbers correspond to those in Table 1. Black, dotted lines indicate ΔT values of -1 and 1°C. Refer to Methods sections for details on the bee preference function.

**Fig 7:**
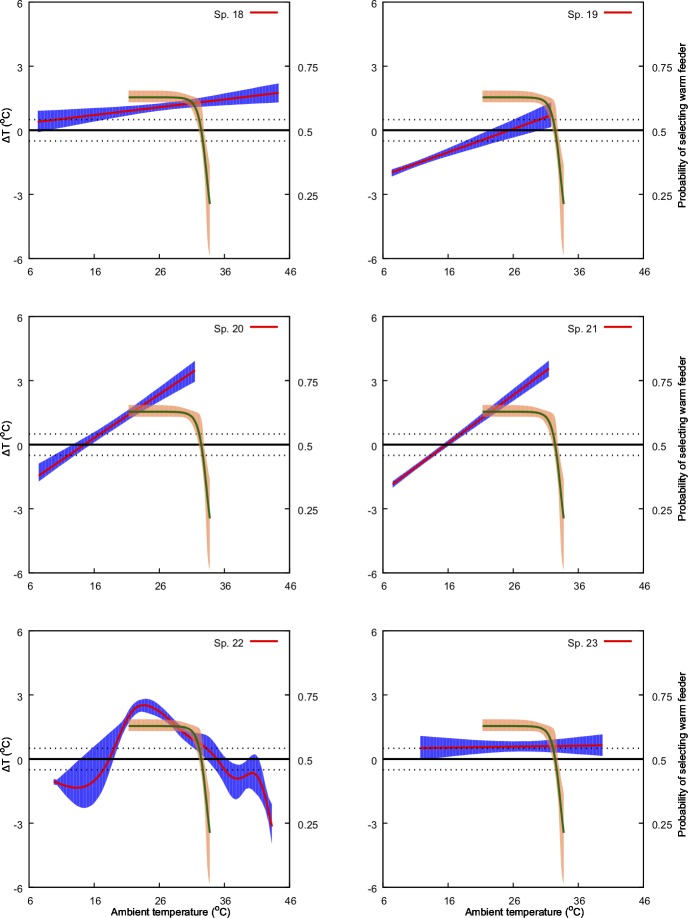
Predicted effect of ambient temperature (*x-*axis) on ΔT (primary *y-*axis: left-hand side) and on bee preference for warmer nectar reward (secondary, *y*-axis: right-hand side). The model for ΔT is represented by the solid red line along with its 95% confidence region (shaded blue region). Solid green line represents the preference model along with its 95% confidence region (shaded orange region). Species numbers correspond to those in Table 1. Black, dotted lines indicate ΔT values of -1 and 1°C. Refer to Methods sections for details on the bee preference function.

**Fig 8:**
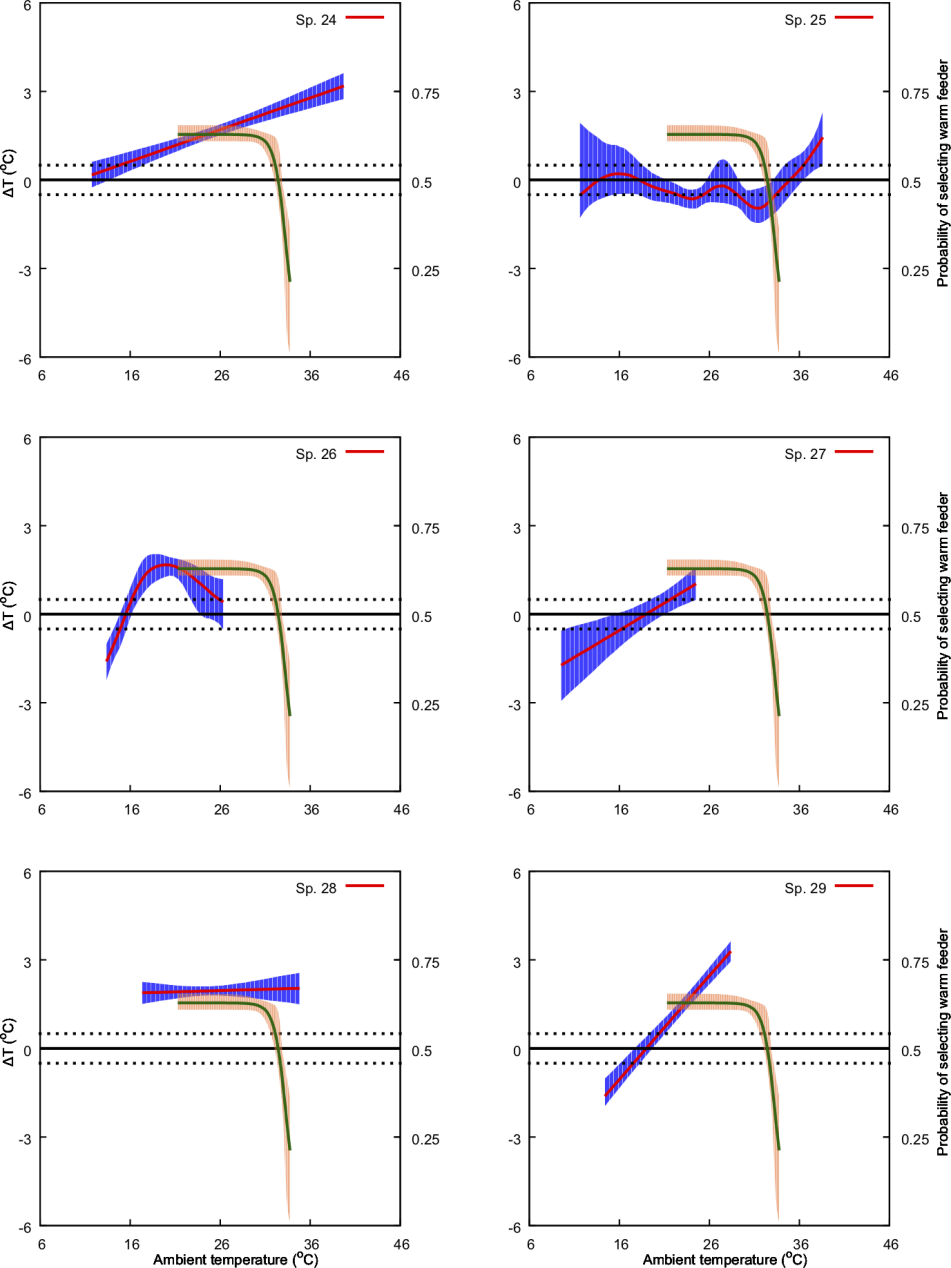
Predicted effect of ambient temperature (*x-*axis) on ΔT (primary *y-*axis: left-hand side) and on bee preference for warmer nectar reward (secondary, *y*-axis: right-hand side). The model for ΔT is represented by the solid red line along with its 95% confidence region (shaded blue region). The solid green line represents the preference model along with its 95% confidence region (shaded orange region). Species numbers correspond to those in Table 1. Black, dotted lines indicate ΔT values of -1 and 1°C. Refer to Methods sections for details on the bee preference function.

**Fig 9:**
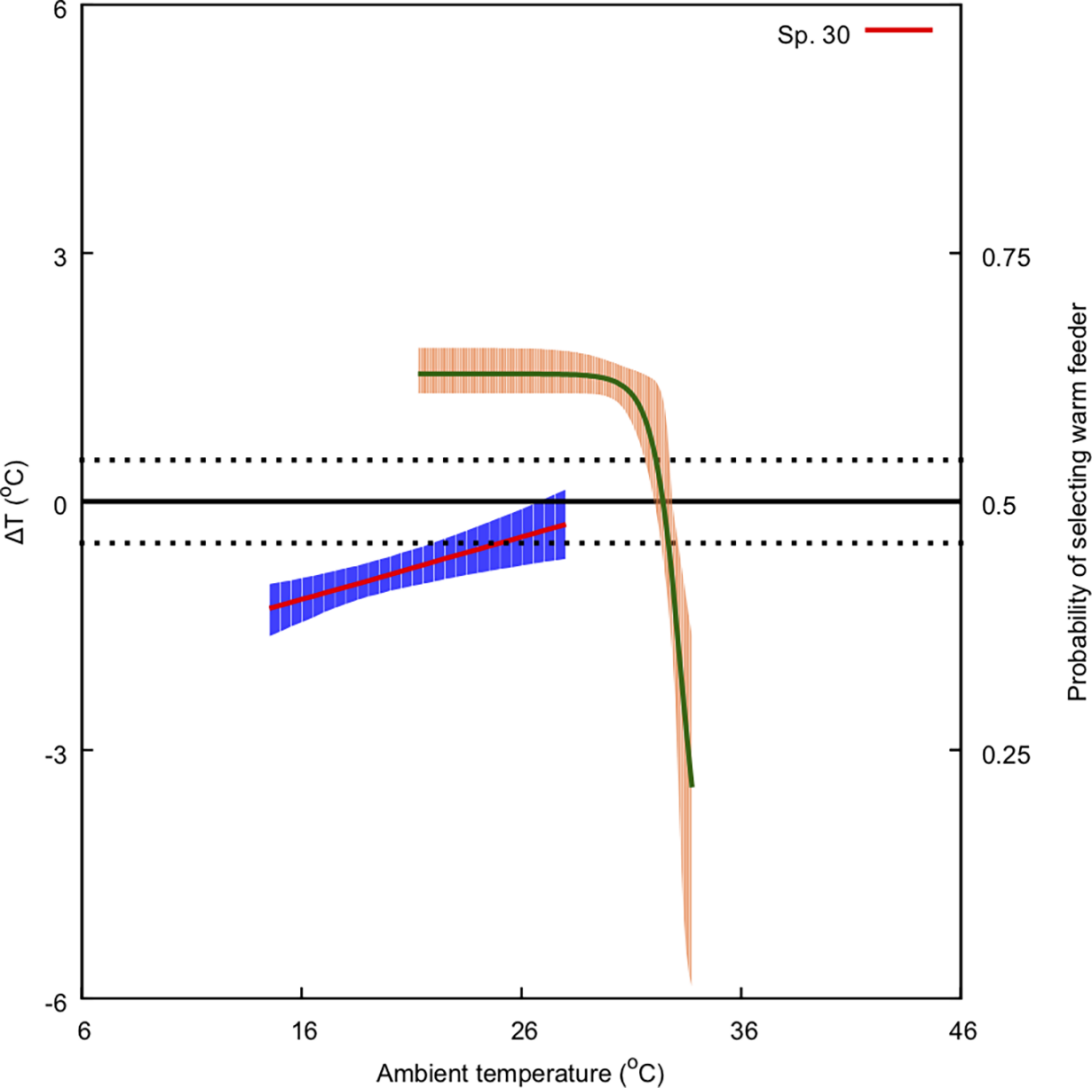
Predicted effect of ambient temperature (*x-*axis) on *ΔT* (primary *y-*axis: left-hand side) and on bee preference for warmer nectar reward (secondary, *y*-axis: right-hand side). The model for *ΔT* is represented by the solid red line along with its 95% confidence region (shaded blue region). The solid green line represents the preference model along with its 95% confidence region (shaded orange region). Species numbers correspond to those in Table 1. Black, dotted lines indicate *ΔT* values of -1 and 1°C. Refer to Methods sections for details on the bee preference function.

We found a non-significant relationship between ambient temperature and *ΔT* for four of the 30 species in our experiments. For the remaining species, we identified either a significant linear positive (13 samples), linear negative (3 samples) or a non-linear relationship (11 samples) between the predictor and the response variable at *α* = 0.05 (Table 1, Figs 4–9). Results of the individual regression analyses are presented in Table 1.

**Fig 10:**
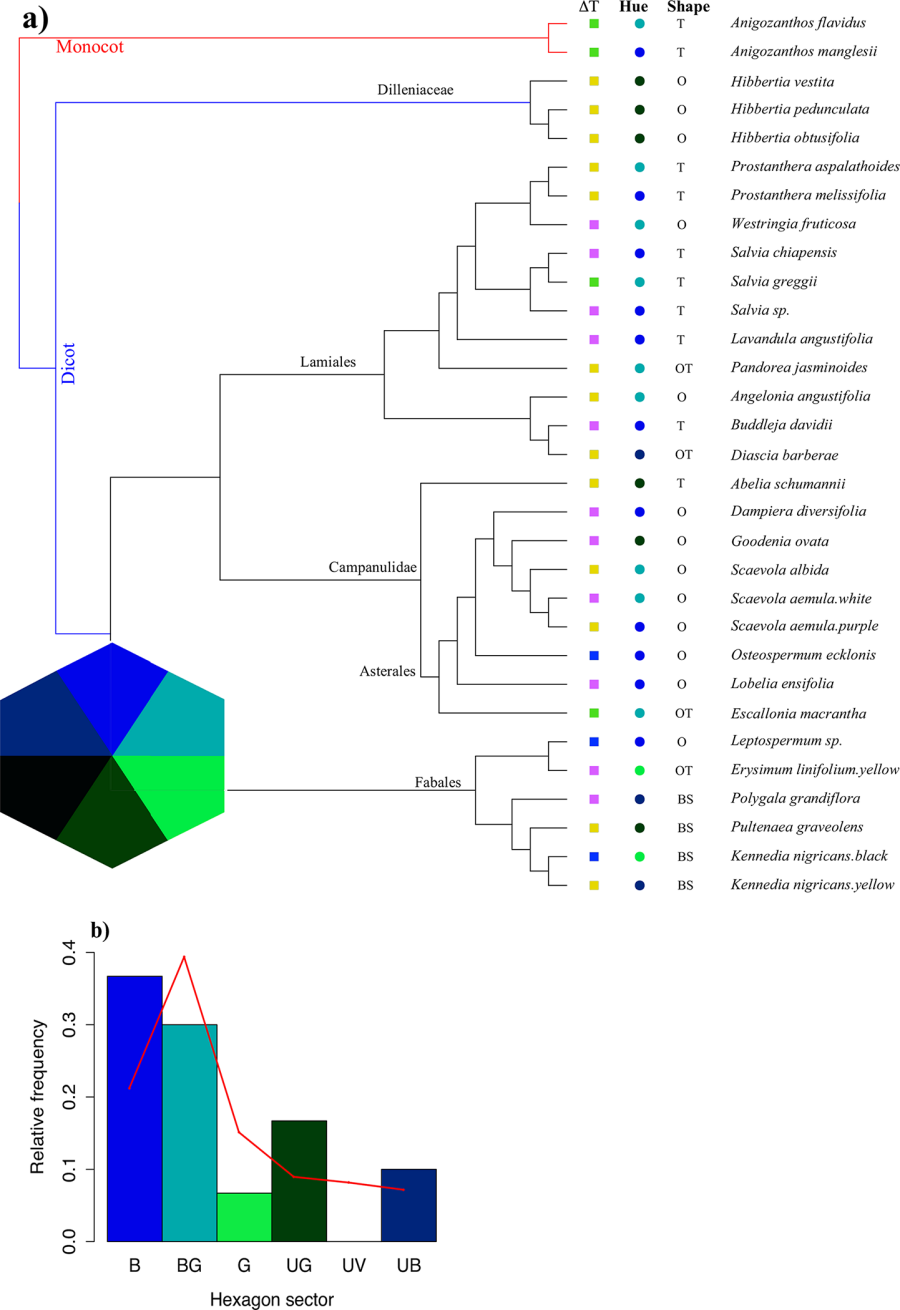
**a**) A representative phylogenetic distribution of flowering plants in our sample following the method of [84] and [85]. A square (solid) represents the relationship between *ΔΤ* and ambient temperature i.e. NS (non-significant, green), NL (non-linear, magenta), LN (linear negative, blue), LP (linear positive, yellow), and a solid circle represents the hue of the flower colour distribution in bee hexagon colour space following [74]. The colours of these symbols are meant only to be distinguishable and to indicate an approximate radial angle (inset hexagon) consistent with current knowledge of bee colour processing. The short abbreviations represent flower shape (see detail in Table 1 Material and Method section). Frequency of ‘hue’ category uses definitions for bee pollinator perception as defined for the hexagon colour space [74]. **b)** The histogram represents the hexagon sector of our sample data, whereas the red line represents the global pattern of flower colour distribution in each hexagon sector [32,33,86]. Hue = Hue in hexagon sector, shape = flower shape.

Among the sampled flowers we classified four main morphologies: boat shape (BS), open (O), open tubular (OT), tubular (T) (Table 2). The colours of the sampled flowers were classified as *BLUE* (G), *BLUE-GREEN* (BG), *GREEN* (G), *UV-GREEN* (UG), *ULTRAVIOLET* (UV) and *UV-BLUE* (UB) in the colour hexagon space for bee perception. The frequencies of the observed colour categories are graphically summarised in Fig 10B.

**Table 2:** Cross-tabulation of sampled flowers by type and shape (upper half), and by type and hexagon sector (lower half). Data expressed as a frequency of observations. Non-significant relationship between the variables (NS), non-linear relationship between predictor and response variable (NL), negative linear relationship (LN), positive linear relationship (LP). Shape of the flower abbreviated as Boat shape (BS), Open (O), Open tubular (OT), tubular (T) whereas flower colours in hexagon colour space are abbreviated as *BLUE* (G), *BLUE-GREEN* (BG), *GREEN* (G), *UV-GREEN* (UG), *ULTRAVIOLET* (UV) and *UV-BLUE* (UB) as defined by [74].

Flower type Flower shape	NS	NL	LN	LP	Shape frequency
BS	0.000	0.033	0.033	0.067	*0*.*133*
O	0.000	0.167	0.067	0.200	*0*.*433*
OT	0.033	0.033	0.000	0.067	*0*.*133*
T	0.100	0.133	0.000	0.067	*0*.*300*
Type frequency	*0*.*133*	*0*.*367*	*0*.*100*	*0*.*400*	
**Flower colour**					Colour frequency
B	0.033	0.200	0.067	0.067	*0*.*367*
BG	0.150	0.100	0.000	0.200	*0*.*450*
G	0.000	0.091	0.091	0.000	*0*.*182*
UB	0.000	0.111	0.000	0.222	*0*.*333*
UG	0.000	0.167	0.000	0.667	*0*.*833*
Type frequency	*0*.*133*	*0*.*367*	*0*.*100*	*0*.*400*	

We found no correlation between temperature type *ΔT* and shape (*χ*^*2*^ = 9.44, df = 9, P = 0.397), or between temperature type *ΔT* and flower colour (*χ*^*2*^ = 16.5, df = 12, P = 0.171) for our samples. Cross-tabulation between these categories is provided in Table 2.

### Bee preference for warm nectar reward modelling

To interpret the modulation of *ΔT* by flowers it is important to understand bee preferences for flower temperature. We thus constructed a model of bee temperature preferences using data for the Australian native bee *T*. *carbonaria* [52].

The three-parameter non-linear model selected to model the preference data (Eq 1) provided a better fit than a GLMM to the preference data (AIC_3-log_: -93.3, AIC_GLMM_: 737.0). Moreover, the non-linear, three-parameter model provided a better fit to the observations than a similar model with two parameters (log-likelihood ratio = 35.54, P < 0.0001).

Coefficients describing the selected non-linear model and their 95% confidence intervals are: A = 0.629 (0.609, 0.655), B = 33.3 (33.0, 34.1) and C = -0.664 (-1.19, -0.178). The model is plotted in (Fig 3) along with the original data from [52].

We used this model (Fig 3) in the temperature range 16–36° C to interpret our results on flower temperature variation and native bee preferences in Figs 4–9.

### Summary of results based on phylogenetic distribution of species

**Fig 11:**
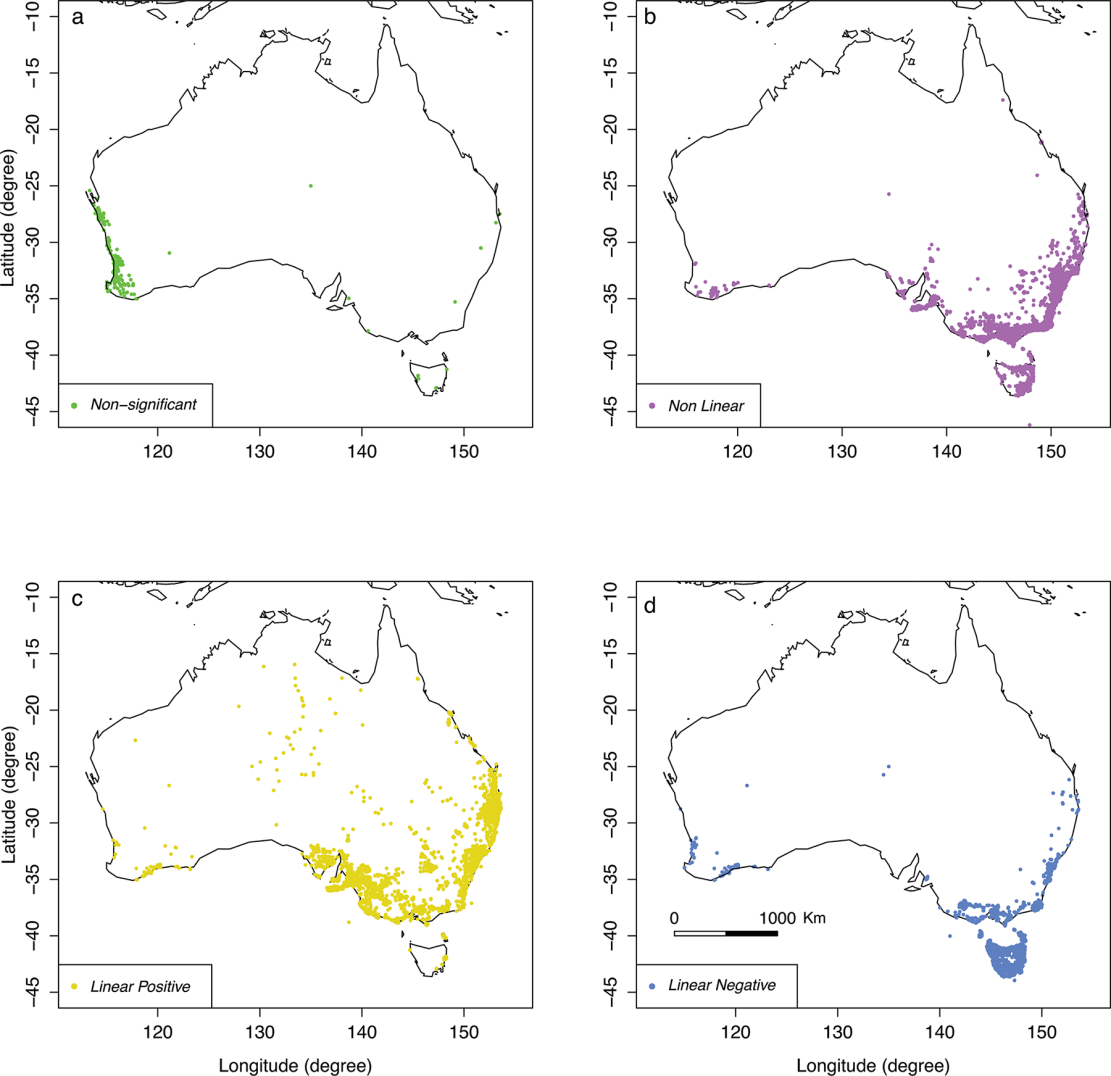
Distribution of measured flower samples in Australia based on type of relationship (details on Table 1) between *ΔΤ* and ambient temperature. a) Non-Significant (NS) flowering plants species distribution, b) Non-Linear (NL) flowering plants species distribution, c) Linear-Positive (LP) flowering plants species distribution, or d) Linear-Negative (LN) flowering plants species distribution. **Data sources:** all the species data co-ordinates have been downloaded from the Global Biodiversity Information Facility (GBIF) (https://www.gbif.org) using the ‘dismo’ package and plotted using “maps” packages in R Version 1.1.423, 2017 [78].

Our sample includes 15 plant families of flowering plants and 30 species (Figs 1 & 10A, Table 1) with different flower shapes and colours (Fig 1, Table 1) to understand potential temperature variation in different petal surfaces (Figs 4–9). We plotted representative the phylogenetic distribution of our plant species, *ΔΤ*, ambient temperature, flower shape, and hue (Fig 10A & 10B) to explore the relationships between the data and the different species’ floral traits. Our results suggest that flowering plants may modulate their temperature, but different species may do this in different ways. We also plotted our sample species distributions within Australia using Global Biodiversity Information Facility [83] data based on *ΔΤ* and ambient temperature classified as in Table 1 (Fig 11).

## Discussion

We identified different types of relationship between ambient temperature and *ΔT* distributed among our sample species. These relationships include: i) non-significant (NS) change (null expectation), ii) non-linear (NL), iii) linear negative (LN) and iv) linear positive (LP) (Table 1, Figs 4–9). We found a non-significant (NS) relationship with flower temperature with ambient temperature (*ΔT*) for 4 samples, non-linear (NL) for 11 samples, linear (LN) for 3 samples and linear positive (LP) for 13 samples (see Table 1, 30 plant species with 31 samples). We found no significant correlation between temperature modulation type group and/or either colour or shape in our sample species. However, the species *Kennedia nigricans* which is represented by two samples (sp4: part 4a & 4b) in our analysis–since it has two distinctly different coloured petal regions–can, on a single flower, present different modulation strategies associated with its differently coloured parts. Our results suggest that complex relationships between flower temperature and ambient temperature are indeed possible.

Previous studies have shown that important pollinators like bumblebees prefer warmer flowers and nectar [28,51], and in Australia the stingless bee *T*. *carbonaria* prefers warmer flowers when the ambient temperature is cool, but may switch preferences when ambient temperature is above 30° C [52]. Corbet et al [75] suggested that small male bees prefer cooler flowers when the ambient temperature exceeds a temperature that a small bee can cope with (44° C), supporting the results of [87] and [52]. However, our understanding of the relationships between flowers and ambient temperature for plant-pollinator interactions is rather limited. In the current study we used behavioural data from Australian native stingless bees [52] to inform our analysis of how, and potentially why, different native and non-native naturalized flowers modulate temperature. Stingless bees have been observed as a flower visitor to all the plant species with in our study [88] and are known pollinators for serval of the plant species in our samples [35,89]. Specifically, we plotted the frequency of bees choosing warmer nectar [52] against the observed effect of ambient temperature on *ΔT* for the different flowers considered in our study (Fig 4–9). Our results show that plant species like *Hibertia vestita* (sp20) and *H*. *obtusifolia* (sp21) (Fig 7) respond to increasing ambient temperature in a simple way, with a linear increase in petal surface temperature. Other flowers like *Anigozanthos flavidus* (sp8) and *Escallonia macrantha* (sp23) have temperatures that do not change in a way that is significantly different to ambient temperature (Table 1, Figs 5 & 7). However, some plant species do modulate temperature in a complex way that would appear suited for the temperature preference of pollinating bees. For example, *Erysimum linifolium* (sp13) and *Polygala grandiflora* (sp15) have a non-linear (NL) relationship between *ΔT* and ambient temperature (Table 1, Fig 6) that resembles the bee temperature preference pattern. Additionally, *Leptospermum* sp. (sp6) and *Osterospermum ecklonis* (sp16) have a linear negative relationship between *ΔT* and ambient temperature (Table 2, Figs 5 & 6).

Our results indicate that different species of plant may use a variety of mechanisms to modulate flower temperature which may have consequences when offering thermal rewards to potential pollinators. The contrasting temperature profiles of the different plants suggests that species like *H*. *vestita* (sp20) and *H*. *obtusifolia* (sp21) appear to lack a cooling mechanism. They may therefore be less attractive to bee pollinators in a scenario where climatic conditions change such that the ambient temperature shifts more frequently above 30° C. This may negatively impact on the flowers’ selection in an evolutionary sense. In contrast, species like *Westringia fruticosa* (sp2), *E*. *linifolium* (sp13) and *Buddleja davidii* (sp23) (Figs 4, 6 & 7) do appear to modulate their temperature in a way consistent with bee preferences, suggesting that a likely fitness benefit might be achieved in a warming climate. Interestingly, the distribution of plants with particular warming or cooling profiles appears clustered in certain regions of Australia (Fig 11). This suggests the possibility of local adaptation. To fully test this observation would require larger sampling worldwide than our study encompasses. We thus encourage future research to build on our experimental framework to enable more comprehensive understanding of the complex worldwide plant-pollinator interactions in different climatic conditions. In particular, future work could focus on how different conditions like wind, relative humidity, and or direct solar exposure may also affect flower temperature modulation at a given ambient temperature. Some effects like heat load and the potential for evaporative cooling by flowers may be greater in direct sunlight, depending on wind (convective cooling), humidity and the plant’s water content. To test such effects however, it may be most effective to concentrate on model plant species like *Arabidopsis thaliana* [90,91], Sacred Lotus [65,92], and/or Snapdragon [56] where great control over the many factors potentially regulating temperature are easier to monitor. Mapping such findings to the broad scale, preliminary survey we report here, should assist us to gain insight into how flowering plants might deal with changing climates. However, given the diversity of flowering plants in nature, it would remain important also to further test more non-model species.

We found no significant relationship between floral temperature modulation category and other visual characteristics of the flowers such as their colour and shape. None of the temperature modulation categories have a colour or shape more frequent than expected by chance (Tables 1–2, Figs 4–9), although some shape or colour combinations were not present in some temperature categories. For example, UG flowers are absent from the LN and NS temperature categories, and the latter category also lacks boat-shaped (BS) and open (O) flowers. A larger sampling effort across the continent is required to test if the colour/temperature category and shape/temperature category combinations that were not observed are indeed rare, or were not observed in our sampling simply by chance. Another possible explanation for the apparent independence of colour and shape from temperature categories, could be that these characteristics are not directly involved in thermoregulation, and other petal characteristics such as nano-texturing and petal orientation are more important for this role as proposed by [30,59].

Flowers in cold environments have recently been observed to have higher temperatures than the ambient temperature [53,54]. For example, the subantarctic megaherbs *Pleurophyllyum speciosum* exhibited higher leaf and floral temperatures than ambient temperature (leaves 9° C higher, inflorescence 11° C higher than ambient temperature) [54]. Some studies suggest that flowering plants regulate temperature based on ambient temperature [65,93]. For example, Sacred Lotus (*Nelumbo nucifera*) maintain the receptacle temperature between 30–36° C at the time of anthesis by increasing temperature at night but decreasing it during the day [65,92]. Grant et al [94] suggested that alternative oxidase (AOX) protein is responsible for the heat regulation in the floral petal, stamen and receptacle in Sacred Lotus. Other studies demonstration that flower petal temperature may be influenced by petal surface microstructure [30,56,59].

Temperature changes may affect plant fitness. For example, [64] experimentally showed that long exposure of a floral organ at constant ambient temperature may damage the petal, pollen and gynoecium. Recently, [95] also suggested that frequent heat waves would decrease the reproductive output of flowering plants. Our study shows that flowering plants in Australia may use a range of mechanisms to modulate temperature with implications for changing environmental conditions (Figs 4–9), however we did not find any simple relationship between flower shape and its response to ambient temperature (Table 1). Thus, we might expect plants to use different mechanisms in a complex fashion to protect their flowers from heat as suggested in previous studies [30,59,96–98]. For example, some flowers are shaped as a paraboloid antenna, a cup shape, that focuses radiation into gynoecium having a positive effect on pollination, fertilization and fruit development [30,59,99]. In a study of 101 herbaceous species of tropical submontane and savanna plants at Mt. Kilimanjaro, Tanzania [100], it was suggested that changes in temperature may affect the future plant community assembly due to the severity of plant adaptation in the different environments. The Tanzanian study shows that submontane plant species’ survival, growth and reproduction improves in cooler environments, whilst savanna plants can survive equally well in either submontane or savanna environments [100]. Thus, changing climatic conditions may create a reproductive barrier to certain plant species (Fig 11), although the effects on pollination and subsequent plant fitness requires further study. Interestingly, in our study we observed that some flowers species (*Salvia* sp., *Dampiera diversifolia*, *Lobelia ensifolia*, *Scaevola albida*, *Hibbertia* spp., *Prostanthera aspalathoides*) wilted when the ambient temperature exceeded 36° C, suggesting that temperature modulation may have a very immediate and direct effect on some plant species’ reproduction–a topic for future research. If increasing temperature does affect floral organs by damaging flower parts, this would immediately interfere with reproductive success by preventing pollinator access to the stigma. Such major temperature-dependent disruptions to plant structure would even have consequences for the effectiveness of new technology including artificial plant-pollinators such as ‘Robo bees’ [101].

Our study shows a range of plasticity of the floral petal surface temperature with respect to ambient temperature. An understanding of this variation is vital to learning which types of flower are most vulnerable to changing environmental conditions. Several studies have shown that mismatch of flowering and pollinator emergence times affects the reproductive activity of flowering plants that depend solely on insect vectors for cross-pollination [2,7,11,19,102]. For example, [7,103] found that when a plant species flowered earlier than usual, the result was reduced seed set during a warmer spring, relative to cooler years. This suggests asynchrony between two bee-pollinated flowering species and bee emergence times with climate warming. The recent FAO [44] and IPBES [47] reports reveal that the economic impact of plant pollination by insects for food production is estimated to be within a range of 235–577 billion US$/year, although currently the potential effects of changing climatic conditions on such a resource are poorly understood [44]. Our study on a range of plant species shows some plants appear to modulate temperature dynamically, and that this behaviour may be regional (Fig 11). This may mean that there is a level of plasticity in at least some plant pollinator systems to counter changing climatic conditions, at least within a certain range. Thus, further work on floral temperature variation around the world would be valuable to map, understand, and help us to manage, important plant pollinator resources.

## Supporting information

S1 Table Supporting_File_S1_RAW_DATA. Raw data of Flower temperature measurement is in .csv format. Details of each file provided in README file.
(ZIP)
