# Correction: Pollination in a new climate: Assessing the potential influence of flower temperature variation on insect pollinator behaviour

**DOI:** 10.1371/journal.pone.0203153

**Published:** 2018-08-23

**Authors:** The PLOS ONE Staff

Fig 2 is incorrect. The authors have provided a corrected version here. The publisher apologizes for the error.

**Fig 2:**
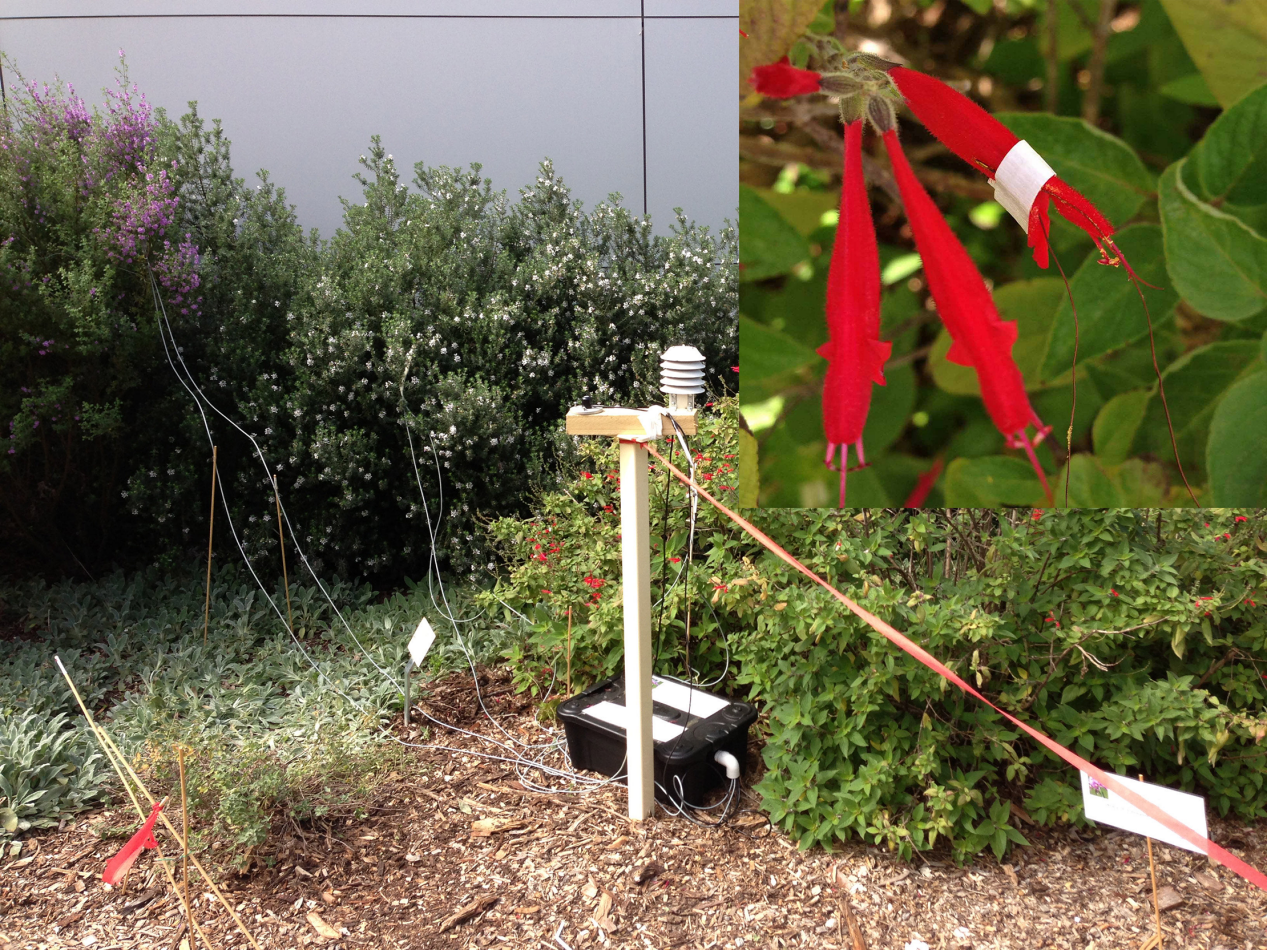
Equipment set up in the field to collect flower temperature readings. Inset shows the TSM sensor attached to a petal surface.
